# Baseline Biomarkers Associated with Early Anatomical Response After Faricimab Loading Therapy in Treatment-Naïve Neovascular Age-Related Macular Degeneration

**DOI:** 10.3390/biomedicines14071590

**Published:** 2026-07-16

**Authors:** Eisuke Ikeuchi, Akiko Miki, Toshiki Oka, Maya Kishimoto-Kishi, Makoto Nakamura

**Affiliations:** Department of Surgery, Division of Ophthalmology, Graduate School of Medicine, Kobe University, 7-5-2 Kusunoki-cho, Chuo-ku, Kobe 650-0017, Japan

**Keywords:** faricimab, neovascular age-related macular degeneration, optical coherence tomography, fibrovascular pigment epithelial detachment, subretinal hyperreflective material, *ARMS2*

## Abstract

**Background/Objectives:** We identified baseline factors associated with early anatomical response to faricimab. **Methods:** This single-center retrospective study included 78 treatment-naïve eyes with neovascular age-related macular degeneration (nAMD) receiving three monthly faricimab injections. Eyes with complete resolution of intraretinal and subretinal fluid on optical coherence tomography (OCT) at 16 weeks constituted the fluid-free group, and the remaining eyes were assigned to the persistent-fluid group. Baseline OCT features (including subretinal hyperreflective material [SHRM], pigment epithelial detachment [PED] subtypes, and central retinal and choroidal thickness) and clinical characteristics were compared, and *ARMS2* (rs10490924) and *CFH* (rs800292) were genotyped as an exploratory analysis. Multivariable logistic regression identified associated factors. **Results:** Fifty-five eyes (70.5%) achieved complete fluid resolution. In multivariable analysis, SHRM was independently associated with a favorable early anatomical response, and fibrovascular PED with incomplete early fluid resolution (*p* = 0.019 and *p* = 0.026); in an exploratory model, the *ARMS2* risk allele was associated with incomplete fluid resolution (*p* = 0.008), whereas *CFH* was not. **Conclusions:** In treatment-naïve nAMD, baseline SHRM was associated with a favorable early anatomical response to faricimab, whereas fibrovascular PED and a higher *ARMS2* T-allele count were associated with incomplete early fluid resolution. These characteristic OCT and genetic features may help identify eyes at risk of an incomplete early response.

## 1. Introduction

Neovascular age-related macular degeneration (nAMD) is a leading cause of severe vision impairment in older adults globally [1,2,3]. Intravitreal injections of anti-vascular endothelial growth factor (VEGF) agents are established as the first-line treatment and effectively preserve visual acuity in patients with nAMD [4,5,6,7]. Despite this broad success, considerable interindividual variability in treatment response persists, and some patients exhibit suboptimal anatomical and functional outcomes even with consistent anti-VEGF therapy [8,9,10]. This heterogeneity underscores the need to better understand factors that influence therapeutic efficacy.

Faricimab is a bispecific antibody targeting both VEGF-A and angiopoietin-2 (Ang-2). Pivotal clinical trials have demonstrated the efficacy and safety of intravitreal faricimab (IVF), and accumulating real-world data further support its ability to improve visual acuity and reduce central retinal thickness (CRT) in both treatment-naïve and previously treated patients [11,12,13,14]. Nonetheless, a subset of patients still exhibits incomplete anatomical response after the initial loading phase.

Previous studies have highlighted the clinical importance of early anatomical response to anti-VEGF treatment. In a post hoc analysis of the ALTAIR study, more than 60% of patients without retinal fluid at week 16 during intravitreal aflibercept treat-and-extend therapy achieved the maximum treatment interval of 16 weeks by week 96, compared with fewer than 20% of patients with residual fluid at week 16 [15]. Persistent retinal fluid in the early treatment phase has also been associated with poorer long-term visual outcomes [16]. These findings emphasize the clinical relevance of identifying baseline factors that predict early treatment responsiveness, as the end of the loading phase represents a key decision point at which incomplete response may prompt closer monitoring or treatment modification.

In parallel with imaging biomarkers, genetic susceptibility factors have been implicated in both the pathogenesis of nAMD and interindividual variability in treatment response [17]. Variants in the age-related maculopathy susceptibility 2 (*ARMS2*) gene represent a major genetic risk determinant for nAMD across diverse populations, including Asian populations [18,19]. Complement pathway genes, including complement factor H (*CFH*), also contribute to disease susceptibility [19]. Genetic studies of anti-VEGF therapy, however, have produced inconsistent associations across studies, treatment agents, and outcome definitions [20,21], and these data have been derived predominantly from cohorts treated with ranibizumab or aflibercept.

Data on baseline predictors of early treatment response to faricimab in treatment-naïve nAMD remain limited. We therefore evaluated baseline factors associated with early anatomical response after the loading phase of faricimab and, as an exploratory analysis, examined two candidate polymorphisms. The aim of this study was to identify clinically assessable baseline factors that may predict the early anatomical response to faricimab loading therapy and thereby support individualized monitoring.

## 2. Materials and Methods

### 2.1. Study Design and Ethical Approval

This single-center retrospective observational study was conducted at Kobe University Hospital. The study adhered to the tenets of the Declaration of Helsinki and the ARVO Statement on Human Subjects. The study protocol was approved by the Institutional Review Board of Kobe University Hospital (approval no. B250202; approved on 26 November 2025). Because this was a retrospective study using residual biological samples, the Institutional Review Board waived the requirement for written informed consent for the present study, and patients were given the opportunity to opt out through public disclosure of the study information. The genetic (DNA) samples analyzed were residual samples originally collected in a previous study, for which written informed consent—including consent for genetic analysis—had been obtained from all participants under a separate study protocol approved by the Institutional Review Board of Kobe University Hospital (approval no. B230032).

### 2.2. Participants

Consecutive patients with treatment-naïve nAMD who received their first intravitreal faricimab (IVF; 6.0 mg/0.05 mL) between May 2022 and October 2024 were retrospectively identified from the medical records. All included eyes had completed a loading phase of three consecutive monthly IVF injections, administered at baseline and at weeks 4 and 8, and were followed for at least 4 months after the initial injection. No additional intravitreal injection was administered between the third loading injection (week 8) and the 16-week assessment; thus, the 16-week endpoint was evaluated 8 weeks after the third injection. Eyes were excluded if they had other retinal diseases that could affect macular anatomy or visual acuity (including diabetic retinopathy and retinal vein occlusion), high myopia (spherical equivalent ≤ −6.0 diopters), submacular hemorrhage of ≥2 disc diameters involving the fovea, prior vitrectomy, prior photodynamic therapy, or prior anti-VEGF treatment.

### 2.3. Outcome Definitions and Rationale for Primary Endpoint

The primary endpoint was early anatomical response at 16 weeks (8 weeks after the third injection), defined as the absence of both intraretinal fluid (IRF) and subretinal fluid (SRF) on optical coherence tomography (OCT) after three monthly loading IVF injections. Eyes with IRF and/or SRF at 16 weeks were assigned to the persistent-fluid group and the remaining eyes to the fluid-free group. These group labels indicate early anatomical (fluid) status at week 16.

### 2.4. Imaging and OCT Parameters

Fluorescein angiography and indocyanine green angiography were performed using the Spectralis HRA2 (Heidelberg Engineering GmbH, Heidelberg, Germany), and macular OCT was performed using the Spectralis OCT system (Heidelberg Spectralis OCT; Heidelberg Engineering GmbH, Heidelberg, Germany), for the diagnosis and subtyping of nAMD according to the Japanese age-related macular degeneration (AMD) diagnostic criteria [22]. A 6-line macular radial scan and a 31-line horizontal raster scan with enhanced depth imaging (EDI) through the fovea were used to evaluate exudative changes (including IRF and SRF) and to measure OCT parameters. CRT was manually measured from the outer surface of the neurosensory retina to the inner surface of the retinal pigment epithelium (RPE) at the foveal center. Central choroidal thickness (CCT) was manually measured from the outer surface of the RPE to the inner surface of the sclera at the foveal center on EDI-OCT images [23,24]. The presence of subretinal hyperreflective material (SHRM) and the presence and subtype of pigment epithelial detachment (PED), classified as serous PED (>2 disc diameters), fibrovascular PED (>3 disc diameters), or hemorrhagic PED (>3 disc diameters), were determined as described previously [9,25]. SHRM was defined as hyperreflective material located between the neurosensory retina and the RPE on OCT. OCT images were assessed by a single retinal specialist. To assess grading reliability, a second retinal specialist, masked to group assignment and clinical outcome, independently regraded the presence of SHRM and of fibrovascular PED in all eyes; inter-observer agreement was quantified using Cohen’s kappa (κ). Best-corrected visual acuity (BCVA) was measured as decimal visual acuity with a Landolt C chart and converted to the logarithm of the minimum angle of resolution (logMAR) for analysis. OCT and clinical assessments were performed at baseline, at each loading visit (weeks 4 and 8 after the initial injection), and at the 16-week assessment; baseline and week-16 data were used for the present analysis.

### 2.5. Genetic Analysis

Residual genomic DNA that had been extracted and stored in a previous study was used for genetic analysis. Single-nucleotide polymorphisms (SNPs) in the *ARMS2* gene (A69S, rs10490924) and the *CFH* gene (I62V, rs800292) were genotyped using TaqMan technology (Thermo Fisher Scientific, Waltham, MA, USA) according to the manufacturer’s instructions. Residual DNA was available for 63 of the 78 eyes (44 in the fluid-free and 19 in the persistent-fluid group), which constituted the genetic analysis set, and genotyping was successful for all analyzed samples (call rate 100%). *ARMS2* rs10490924 and *CFH* rs800292 were both analyzed using an additive logistic regression model based on allele count (T-allele count for *ARMS2* and A-allele count for *CFH*).

### 2.6. Statistical Analysis

Continuous variables are presented as mean ± standard deviation. The Wilcoxon signed-rank test was used to compare pre- and post-treatment measures within groups. For comparisons between the fluid-free and persistent-fluid groups, the Mann–Whitney U test was used for continuous variables and a two-sided Fisher’s exact test for categorical variables. Variables with *p* < 0.10 in the comparison between the fluid-free and persistent-fluid groups were considered candidates for the multivariable model; to avoid overfitting given the limited number of events, Model 1 (clinical/OCT model) was restricted to two OCT imaging biomarkers, and Model 2 additionally included the *ARMS2* T-allele count as an exploratory variable. A *p* value < 0.05 was considered statistically significant, and all tests were two-sided. Statistical analyses were performed in SPSS v29 (IBM Corp., Armonk, NY, USA); the Firth penalized logistic regression sensitivity analysis was performed in R v4.6.1. No sample-size calculation was performed in advance; all consecutive eligible eyes during the study period were included. To verify the stability of the estimates given the limited number of events, Model 1 was additionally re-estimated using Firth’s penalized (bias-reduced) logistic regression as a sensitivity analysis. The multivariable model was also re-estimated using the second reader’s gradings as a further sensitivity analysis.

## 3. Results

### 3.1. Baseline Characteristics

In total, 82 treatment-naïve nAMD eyes completed three monthly faricimab loading injections; four were excluded—two because of less than 16 weeks of follow-up and two because they met the exclusion criteria—leaving 78 eyes of 78 patients for analysis. Based on early anatomical response at 16 weeks, 55 eyes (70.5%) were classified as the fluid-free group and 23 eyes (29.5%) as the persistent-fluid group. Baseline clinical characteristics are summarized in Table 1. There were no statistically significant differences between groups in age, sex, baseline BCVA, baseline CRT, or baseline CCT. Baseline SHRM was significantly more frequent in the fluid-free group than in the persistent-fluid group (67.3% vs. 39.1%, *p* = 0.026). Fibrovascular PED >3 disc diameters tended to be more frequent in the persistent-fluid group (26.1% vs. 7.3%, *p* = 0.056). The proportion of polypoidal choroidal vasculopathy (PCV) and the macular neovascularization (MNV) subtype distribution did not differ significantly between groups. Inter-observer agreement for the qualitative OCT features was almost perfect (Cohen’s κ = 0.95 for SHRM and 0.94 for fibrovascular PED).

### 3.2. Functional and Anatomical Outcomes at Week 16

In the overall cohort, mean BCVA significantly improved at 16 weeks (from 0.26 ± 0.38 to 0.17 ± 0.39 logMAR; *p* < 0.001). Mean CRT and CCT also significantly decreased compared with baseline (CRT 335.4 ± 122.9 to 195.3 ± 88.7 μm, *p* < 0.001; CCT 202.0 ± 100.6 to 184.1 ± 99.0 μm, *p* < 0.001) (Table 2). In the fluid-free group, BCVA, CRT, and CCT all improved significantly (all *p* < 0.001). In the persistent-fluid group, mean CRT decreased significantly (353.7 ± 134.3 to 232.9 ± 114.8 μm, *p* < 0.001), whereas mean CCT showed only a borderline change (217.3 ± 121.4 to 197.3 ± 106.1 μm, *p* = 0.061), and mean BCVA improved significantly (0.33 ± 0.48 to 0.27 ± 0.53 logMAR, *p* = 0.024).

### 3.3. Genetic Analysis (Exploratory)

Residual DNA was available for genotyping in 63 of the 78 eyes. The genotype distributions and allele frequencies are shown in Table 3. Because the *ARMS2* G/G genotype was absent in the persistent-fluid group, analyses were based on allele frequencies rather than genotypes. The minor (T) allele frequency of *ARMS2* rs10490924 was significantly lower in the fluid-free group than in the persistent-fluid group (51.1% vs. 81.6%, *p* = 0.001, allelic chi-square test; odds ratio [OR] per T-allele 4.24, 95% confidence interval [CI] 1.69–10.65). The *CFH* rs800292 A-allele frequency did not differ between groups (28.4% vs. 28.9%, *p* = 1.000).

### 3.4. Multivariable Logistic Regression

To identify factors associated with the 16-week treatment response, variables with *p* < 0.10 in the comparison between the fluid-free and persistent-fluid groups were considered for the multivariable logistic regression model. Three variables met this threshold—SHRM (*p* = 0.026), fibrovascular PED (*p* = 0.056), and axial length (*p* = 0.084); to limit the number of predictors given the limited number of events, only the two OCT imaging biomarkers (SHRM and fibrovascular PED) were entered, and axial length, which showed the weakest association, was not included. In Model 1 (clinical/OCT only, n = 78), baseline SHRM was independently associated with favorable early anatomical response (OR 0.28, 95% CI 0.10–0.81, *p* = 0.019), and fibrovascular PED was independently associated with incomplete early fluid resolution (OR 5.27, 95% CI 1.22–22.71, *p* = 0.026) (Table 4). In a sensitivity analysis using Firth’s penalized logistic regression, these associations were essentially unchanged and remained significant (SHRM: OR 0.30, 95% CI 0.10–0.82, *p* = 0.019; fibrovascular PED: OR 4.81, 95% CI 1.23–20.93, *p* = 0.024), supporting the stability of the estimates. The associations also persisted when the model was re-estimated using the independent second reader’s gradings (SHRM: OR 0.25, 95% CI 0.08–0.72, *p* = 0.011; fibrovascular PED: OR 6.81, 95% CI 1.40–33.09, *p* = 0.017).

In Model 2, which additionally included the *ARMS2* T-allele count as an exploratory variable, the association of baseline SHRM with favorable response remained significant (OR 0.12, 95% CI 0.03–0.55, *p* = 0.006), and the *ARMS2* T-allele count was associated with incomplete fluid resolution (OR per T-allele 5.52, 95% CI 1.57–19.44, *p* = 0.008). Fibrovascular PED was no longer significantly associated with incomplete fluid resolution in this exploratory model (*p* = 0.293).

## 4. Discussion

In this retrospective study, we identified baseline OCT features associated with early anatomical response to faricimab loading therapy in treatment-naïve nAMD. Approximately one-third of eyes (29.5%) showed persistent IRF and/or SRF after the loading phase, indicating substantial interindividual variability in early response. The principal findings were that baseline SHRM was independently associated with favorable early anatomical response, whereas fibrovascular PED was independently associated with incomplete fluid resolution. As an exploratory genetic analysis, the *ARMS2* rs10490924 T-allele was associated with incomplete early fluid resolution, whereas *CFH* rs800292 showed no significant association. Rather than predicting long-term outcomes, these findings may be more useful for identifying eyes that warrant closer monitoring or earlier consideration of treatment modification after the loading phase, supporting early identification of at-risk eyes in the clinical management of treatment-naïve nAMD.

Consistent with previous faricimab reports [26,27], mean BCVA, central retinal thickness, and central choroidal thickness all improved significantly at 16 weeks compared with baseline in the present cohort (all *p* < 0.001). The proportion of eyes in the persistent-fluid group in the present cohort (29.5%) was slightly higher than in previous Japanese faricimab studies that used the same three-monthly loading regimen and the same assessment timepoint (16 weeks/month 4, i.e., 8 weeks after the third injection). Matsumoto et al. reported a dry macula rate of 79.5% (residual fluid 20.5%) in 40 treatment-naïve eyes [26], and Tanaka et al. reported 77.3% (residual fluid 22.7%) in a prospective cohort of 23 patients [27]. This modest difference may reflect differences in baseline disease-subtype composition between cohorts, as well as the larger sample size of the present study (78 eyes vs. 40 and 23), given that estimates from smaller cohorts are less precise and more susceptible to sampling variation.

SHRM, defined as hyperreflective material located between the neurosensory retina and the RPE, is an OCT-defined finding with pathological correlates, including fibrin deposition, subretinal hemorrhage, lipid exudation, and neovascular tissue, either alone or in combination [28]. In aflibercept-treated cohorts evaluating longer-term treatment response, SHRM has been less prevalent in non-responders, consistent with the present findings [8]. Components of SHRM may reflect VEGF-dependent lesion activity that responds to anti-VEGF therapy. It should be noted, however, that SHRM has been associated with poorer long-term visual prognosis in some longitudinal studies [29]. Because the endpoint of the present study was fluid resolution rather than visual acuity, SHRM may predict early fluid resolution without necessarily indicating a favorable long-term visual outcome; its long-term visual implications therefore warrant separate investigation. The individual components of SHRM have each been reported to be associated with a favorable response to faricimab: MNV types 2 and 3 are associated with anatomical responsiveness to faricimab [30], and a recent multicenter cohort study from the J-CREST Group reported that eyes with pretreatment submacular hemorrhage achieved a significantly higher rate of dry macula after IVF (93.9% vs. 78.4%, *p* = 0.048) [31]. In the present cohort, AMD subtype did not show a significant association with early response, whereas SHRM emerged as an independent predictor. Because type 2 neovascular tissue and subretinal hemorrhage often manifest as components of SHRM on OCT [28], SHRM—as a comprehensive imaging feature encompassing subretinal hemorrhage, fibrin, lipid exudation, and type 2 neovascular tissue—may capture lesion activity more directly than angiographic classification alone and serve as a composite marker of VEGF-dependent lesion activity that responds to faricimab loading therapy. Although the favorable association of SHRM with early anatomical response may at least partly reflect an effect common to anti-VEGF agents in general, as suggested by the reduced prevalence of SHRM in aflibercept non-responders [8], the particularly favorable response of submacular hemorrhage [31] and type 2 macular neovascular tissue [30] to faricimab raises the possibility that dual VEGF-A/Ang-2 inhibition may confer an additional advantage in eyes with these phenotypes. Whether this SHRM-related anatomical benefit is common to anti-VEGF agents in general or partly specific to faricimab needs to be examined in direct comparative studies of faricimab and conventional anti-VEGF agents.

In contrast to a previous report in which baseline serous PED was associated with non-responsiveness to intravitreal aflibercept in treatment-naïve nAMD [9], serous PED was not identified as a predictor of incomplete early fluid resolution in the present cohort of faricimab-treated eyes. This discrepancy may be partly attributable to the low prevalence of serous PED in our cohort (4 of 78 eyes, 5.1%, compared with 12 of 62 treatment-naïve eyes, approximately 19%, in the previous report [9]), but could also reflect a pharmacological difference between aflibercept and faricimab; in particular, the additional Ang-2 blockade of faricimab may help stabilize vascular permeability and reduce serous fluid accumulation under the RPE. This hypothesis warrants further investigation in larger cohorts that include more eyes with serous PED. The opposite associations of SHRM and fibrovascular PED with early anatomical response are biologically interpretable as differences in lesion maturity and VEGF/Ang-2 dependency. SHRM-containing lesions typically contain active, exudative components that depend on VEGF and Ang-2 signaling and respond well to their inhibition. In contrast, fibrovascular PED reflects a more structurally mature, partially fibrotic neovascular complex in which VEGF and Ang-2 signaling are thought to contribute less to fluid persistence. The opposite findings in our cohort—favorable response with SHRM and incomplete fluid resolution with fibrovascular PED—are consistent with this framework. However, this biological interpretation will require confirmation in further experimental studies. To our knowledge, fibrovascular PED has not been specifically evaluated as a baseline predictor of incomplete early fluid resolution after faricimab in treatment-naïve nAMD, and it has not been identified as an independent baseline predictor of anti-VEGF response in treatment-naïve cohorts. These preliminary observations suggest that baseline fibrovascular PED may warrant further evaluation as a candidate risk-stratification biomarker; however, the association was of borderline strength and based on a small number of eyes and requires confirmation in larger cohorts.

The *ARMS2* rs10490924 T-allele was more frequent in the persistent-fluid group and remained associated with early anatomical response in an exploratory multivariable model. *ARMS2* has been consistently identified as a major susceptibility locus for nAMD and has been linked to aggressive disease phenotypes [32,33,34]. Notably, a previous genetic study reported that the *ARMS2* major allele (G) was associated with a higher likelihood of PED regression under anti-VEGF treatment in PCV [34], such that the T risk allele corresponded to poorer PED resolution; this directional consistency lends additional support to the exploratory finding observed in our cohort. In our cohort, fibrovascular PED was more frequent in the persistent-fluid group in univariate analysis but did not remain significant once the *ARMS2* risk-allele count was included in the exploratory model, raising the possibility that the two may capture overlapping aspects of disease phenotype; this interpretation is speculative and requires confirmation. These exploratory findings are preliminary and require validation in larger cohorts.

Taken together, the predictors of early anatomical response identified here appear to depend on markers of lesion activity and maturity rather than on any single angiographic subtype, and combining OCT imaging biomarkers with genetic susceptibility may further refine baseline risk stratification. Confirming these observations—including the exploratory genetic associations—will require a prospective, multicenter study with a uniform post-loading regimen, a pre-registered sample size, standardized OCT acquisition, and masked, independently adjudicated central grading.

### Limitations

This study has several limitations. First, the design was retrospective and single-center, with a small sample size. Second, the genetic analysis was based on a small number of eyes and was therefore exploratory. Third, we did not evaluate long-term functional or anatomical outcomes, although early anatomical response after the loading phase—the focus of this study—is itself a clinically important outcome. Fourth, primary OCT grading was performed by a single reader who was not formally masked; although inter-observer agreement for SHRM and fibrovascular PED was almost perfect (Cohen’s κ = 0.94–0.95), some classification bias cannot be fully excluded; in addition, formal intra-observer agreement was not assessed. Fifth, the study cohort was Japanese, and the generalizability of these findings to other populations remains to be confirmed.

## 5. Conclusions

In treatment-naïve nAMD, baseline SHRM and fibrovascular PED on OCT were independently associated with early anatomical response to faricimab loading therapy, with SHRM predicting favorable response and fibrovascular PED predicting incomplete fluid resolution. In an exploratory genetic analysis, the *ARMS2* rs10490924 T-allele count was also associated with incomplete early fluid resolution. These baseline biomarkers may help identify eyes that warrant closer monitoring or earlier consideration of treatment modification after the loading phase, supporting individualized post-loading clinical management. Larger prospective studies are warranted to validate these findings and to confirm their clinical utility.

## Figures and Tables

**Table 1:** Patient baseline clinical characteristics.

Factor	Fluid-Free Group (n = 55)	Persistent-Fluid Group (n = 23)	*p* Value
Age, mean ± SD (years)	77.0 ± 7.7	75.6 ± 9.6	0.653
Sex (male/female)	34/21	14/9	1.000
Axial length, mean ± SD (mm)	23.94 ± 1.33	23.38 ± 1.12	0.084
Smoking (current or past), n (%)	1 (1.8)	1 (4.3)	0.505
Hypertension, n (%)	29 (52.7)	12 (52.2)	1.000
Diabetes mellitus, n (%)	9 (16.4)	4 (17.4)	1.000
Baseline BCVA, mean ± SD (logMAR)	0.24 ± 0.33	0.33 ± 0.48	0.900
MNV subtype (type 1/type 2/type 1+2/type 3), n	36/4/10/5	16/3/3/1	0.730
PCV, n (%)	17 (30.9)	4 (17.4)	0.272
Pachychoroid, n (%)	19 (34.5)	7 (30.4)	0.797
SHRM, n (%)	37 (67.3)	9 (39.1)	0.026
IRF, n (%)	19 (34.5)	10 (43.5)	0.608
SRF, n (%)	46 (83.6)	20 (87.0)	1.000
Serous PED (>2 DD), n (%)	2 (3.6)	2 (8.7)	0.577
Fibrovascular PED (>3 DD), n (%)	4 (7.3)	6 (26.1)	0.056
Hemorrhagic PED (>3 DD), n (%)	0 (0.0)	0 (0.0)	—
Baseline CRT, mean ± SD (μm)	327.8 ± 118.2	353.7 ± 134.3	0.450
Baseline CCT, mean ± SD (μm)	195.6 ± 91.1	217.3 ± 121.4	0.570

SD = standard deviation; MNV = macular neovascularization; PCV = polypoidal choroidal vasculopathy; SHRM = subretinal hyperreflective material; IRF = intraretinal fluid; SRF = subretinal fluid; PED = pigment epithelial detachment; DD = disc diameter; CRT = central retinal thickness; CCT = central choroidal thickness.

**Table 2:** Clinical outcomes at baseline and at week 16 in the overall cohort and by treatment response group.

Group	Variable	Baseline	Week 16	*p* Value
All patients (n = 78)	BCVA (logMAR)	0.26 ± 0.38	0.17 ± 0.39	<0.001
	CRT (μm)	335.4 ± 122.9	195.3 ± 88.7	<0.001
	CCT (μm)	202.0 ± 100.6	184.1 ± 99.0	<0.001
Fluid-free group (n = 55)	BCVA (logMAR)	0.24 ± 0.33	0.13 ± 0.31	<0.001
	CRT (μm)	327.8 ± 118.2	179.3 ± 70.3	<0.001
	CCT (μm)	195.6 ± 91.1	178.5 ± 96.2	<0.001
Persistent-fluid group (n = 23)	BCVA (logMAR)	0.33 ± 0.48	0.27 ± 0.53	0.024
	CRT (μm)	353.7 ± 134.3	232.9 ± 114.8	<0.001
	CCT (μm)	217.3 ± 121.4	197.3 ± 106.1	0.061

Values are mean ± standard deviation. BCVA = best-corrected visual acuity; CRT = central retinal thickness; CCT = central choroidal thickness.

**Table 3:** *ARMS2* (rs10490924) and *CFH* (rs800292) genotype distributions and allele frequencies by treatment response.

SNP	Genotype/Allele	Fluid-Free Group	Persistent-Fluid Group	OR (95% CI)	*p* Value
*ARMS2* *rs10490924*	G/G, %	29.5	0		
	G/T, %	38.6	36.8		
	T/T, %	31.8	63.2		
	T-allele, %	51.1	81.6	4.24 (1.69–10.65)	0.001 *
*CFH* *rs800292*	A/A, %	9.1	15.8		
	A/G, %	38.6	26.3		
	G/G, %	52.3	57.9		
	A-allele, %	28.4	28.9	0.98 (0.43–2.22)	1.000 *

*ARMS2* = age-related maculopathy susceptibility 2; *CFH* = complement factor H; SNP = single-nucleotide polymorphism; OR = odds ratio; CI = confidence interval. ORs for the allelic comparison are presented per minor allele. * Allelic comparison (chi-square test).

**Table 4:** Multivariable logistic regression analyses for incomplete early fluid resolution after faricimab loading therapy.

Variable	OR	95% CI	*p* Value
***Model 1: Clinical/OCT model***			
SHRM (present vs. absent)	0.28	0.10–0.81	0.019
Fibrovascular PED (present vs. absent)	5.27	1.22–22.71	0.026
***Model 2: Clinical/OCT + exploratory ARMS2 model***			
SHRM (present vs. absent)	0.12	0.03–0.55	0.006
Fibrovascular PED (present vs. absent)	2.32	0.48–11.11	0.293
*ARMS2* rs10490924 T-allele count (additive, 0/1/2)	5.52	1.57–19.44	0.008

*ARMS2* = age-related maculopathy susceptibility 2; CI = confidence interval; OCT = optical coherence tomography; OR = odds ratio; PED = pigment epithelial detachment; SHRM = subretinal hyperreflective material.

## Data Availability

The raw data supporting the conclusions of this article are not publicly available because patient-level data contain potentially identifying information but will be made available by the authors upon request and with the approval of the Institutional Review Board of Kobe University Hospital.
